# Quantitative trait locus analysis of growth and wood density in an interspecific pseudo-backcross population of *Eucalyptus grandis* x *E. urophylla*

**DOI:** 10.1186/1753-6561-5-S7-P30

**Published:** 2011-09-13

**Authors:** ARK Kullan, MM Van Dyk, Nicky Jones, Arnulf Kanzler, Arlene Bayley, AA Myburg

**Affiliations:** 1 Department of Genetics, Forestry and Agricultural Biotechnology Institute (FABI), University of Pretoria, Pretoria, 0002, South Africa; 2 Sappi Forests Research, Shaw Research Centre, PO Box 473, Howick, 3290, South Africa; 3 Sappi Technology Centre, PO Box 6, The Innovation Hub, Lynnwood, Pretoria 0087, South Africa

## Background

F1 hybrids of *E. grandis* and *E. urophylla* are commonly grown for pulp and paper production in clonal plantations in tropical and subtropical regions. Improving tree growth [1]and wood quality [2]are important objectives in eucalypt breeding programmes. The efficiency of selection for these traits can be enhanced by molecular breeding approaches enabled by high-throughput, genome-wide genotyping technologies and the recent completion of a reference genome sequence for *Eucalyptus* (*E. grandis* V1.0, JGI, http://www.phytozome.net). In this context, interspecific hybrid pedigrees are valuable for quantitative trait locus (QTL) dissection in *Eucalyptus* as there is abundant interspecific variation in such pedigrees. Breeding for wood property traits is mainly focussed on increasing pulp yield per hectare, reducing wood specific consumption (WSC) and increasing the efficiency of downstream processing by the pulp and paper industry. In this study, we are characterizing the genetic architecture of growth and wood quality traits in an interspecific pseudo-backcross mapping population of an F1 hybrid of *E. grandis* and *E. urophylla.*

## Methods

A pseudo-backcross pedigree was developed by crossing an F1 hybrid (*E. grandis* x *E. urophylla*, GUSAP1, Sappi Forest Research) clone to non-parental individuals of the two parental species. Diameter (cm) at breast height (DBH) and wood basic density were measured at three years in a set of 305 and 319 progeny of the *E. grandis* and *E. urophylla* backcross families, respectively. The backcross progeny were also genotyped with more than 1700 polymorphic Diversity Arrays Technology (DArT) markers. Framework genetic linkage maps were constructed for the *E. grandis*, *E. urophylla* backcross parents using 139 and 127 selected testcross (1:1) DArT markers distributed across the 11 *Eucalyptus* linkage groups spanning 926 cM and 1100 cM, respectively. The framework maps of the F1 hybrid contained 172 and 154 testcross DArT markers spanning 1061 cM and 1036 cM in the *E. grandis* and *E. urophylla* backcross families, respectively. The average marker interval of the parental framework maps ranged from 5.6 (F1 hybrid in the *E. grandis* backcross family) to 8.0 (*E. urophylla* backcross parent). DBH of the main stem was measured directly for three-year-old backcross progeny. For the assessment of basic density, a wood disk was taken at a height of 1.5 m. The wood disk from each tree was used to determine basic density by the water displacement method. QTL analysis was conducted using Windows QTL Cartographer using composite interval mapping (CIM) and a genome-wide significance threshold of α = 0.05.

## Results and conclusion

A total of 9 QTLs were identified for DBH and 15 QTLs were identified for basic density (Table 1). These explained 3.1 to 9.6% and 3.6 to 13.1%of the phenotypic variation in the *E. grandis* and *E. urophylla* backcross families, respectively**.** Higher numbers of QTL were identified in the F1 hybrid parent (15) compared to the backcross parents (9) congruent with the expected interspecific and intraspecific genetic variation segregating in the backcross families.

Comparative QTL mapping in the F1 hybrid and backcross parents identified common and unique QTL regions in the parental maps. On LG6 and LG10, QTLs for DBH and basic density were detected in both F1 hybrid maps (*E. grandis* and *E. urophylla* backcross families). On LG4 and LG9, QTLs for density were shared between the *E. urophylla* and F1 hybrid parent, while a QTL for DBH on LG10 was shared between the *E. grandis* and F1 hybrid parents. Overall, six unique genomic regions with QTLs for DBH and eleven QTLs regions for basic density were identified in the two backcross families. The lower number of significant QTLs identified for DBH compared to that for wood basic density (Table 1) is consistent with previous QTL reports and genetic studies showing that wood quality traits have higher heritability than growth traits [2,3]. The genetic maps constructed and QTL regions identified in this study provide a resource for identifying markers for molecular breeding of volume growth and wood density in *E. grandis* x *E. urophylla*hybrids, as well as candidate gene identification based on the recently released *E. grandis* reference genome sequence.

**Table 1:** Putative QTL for DBH and basic density identified by CIM in *E. grandis* and *E. urophylla* BC mapping population.

Parent tree	Total number of QTLs identified	Linkage group (LG) number for DBH	Linkage group (LG) number for density	Percentage of variation explained by the DBH QTLs	Percentage of variation explained by the density of QTLs
*E. grandis* backcross parent	3	LG9, LG10	LG2	3.6 to 4.6%	4.8%
*E. grandis* F1 hybrid parent	7	LG4, LG6, LG10	LG1, LG3, LG4, LG10	3.7 to 9.6%	3.1 to 5.5%
*E. urophylla* F1 hybrid parent	8	LG6	LG2, LG4, LG6, LG8, LG9, LG10a, LG10b	7.0%	3.6 to 13.1%
*E. urophylla* backcross parent	6	LG4, LG9, LG10	LG4, LG8, LG9	4.5-5.3%	4.6-7.8%
Total	24	9	15		

QTL, Quantitative trait loci; DBH, Diameter at breast height, CIM, Composite interval mapping
